# Molecular Evaluation of Endometrial Dedifferentiated Carcinoma, Endometrioid Carcinoma, Carcinosarcoma, and Serous Carcinoma Using a Custom-Made Small Cancer Panel

**DOI:** 10.3389/pore.2021.1610013

**Published:** 2021-12-23

**Authors:** Yusuke Kobayashi, Ikumi Kitazono, Toshiaki Akahane, Shintaro Yanazume, Masaki Kamio, Shinichi Togami, Sachio Nohara, Ippei Sakamoto, Seiya Yokoyama, Kazuhiro Tabata, Hiroaki Kobayashi, Akihide Tanimoto

**Affiliations:** ^1^ Course of Advanced Cancer Medicine for Gynecologic Cancer, Kagoshima, Japan; ^2^ Department of Pathology, Kagoshima University Graduate School of Medical and Dental Sciences, Kagoshima, Japan; ^3^ Center for Human Genome and Gene Analysis, Kagoshima University Hospital, Kagoshima, Japan; ^4^ Department of Obstetrics and Gynecology, Kagoshima University Graduate School of Medical and Dental Sciences, Kagoshima, Japan; ^5^ Department of Biomedical Informatics, Mitsubishi Space Software, Amagasaki, Japan

**Keywords:** microsatellite instability, tumor mutation burden, integrated molecular diagnosis, endometrial cancers, solid proliferation

## Abstract

It is often difficult to histologically differentiate among endometrial dedifferentiated carcinoma (DC), endometrioid carcinoma (EC), serous carcinoma (SC), and carcinosarcoma (CS) due to the presence of solid components. In this study, we aimed to categorize these carcinomas according to The Cancer Genome Atlas (TCGA) classification using a small custom-made cancer genome panel (56 genes and 17 microsatellite regions) for integrated molecular diagnosis. A total of 36 endometrial cancer cases with solid components were assessed using IHC, next-generation sequencing (NGS), and the custom-made panel. Among 19 EC cases, six were categorized as MMR-deficient (MMR-d) and eight were classified as having a nonspecific molecular profile. Three EC cases were classified as *POLE* mutation (*POLE*mut)-type, which had a very high tumor mutation burden (TMB) and low microsatellite instability (MSI). Increased TMB and MSI were observed in all three DC cases, classified as MMR-d with mutations in *MLH1* and *POLD1*. Except for one case classified as MMR-d, all SC cases exhibited *TP53* mutations and were classified as p53 mutation-type. SC cases also exhibited amplification of *CCND1*, *CCNE1*, and *MYC*. CS cases were classified as three TCGA types other than the *POLE*mut-type. The IHC results for p53 and ARID1A were almost consistent with their mutation status. NGS analysis using a small panel enables categorization of endometrial cancers with solid proliferation according to TCGA classification. As TCGA molecular classification does not consider histological findings, an integrated analytical procedure including IHC and NGS may be a practical diagnostic tool for endometrial cancers.

## Introduction

Dedifferentiated carcinoma (DC) is a rare endometrial cancer accounting for 2% of all endometrial cancers. It is composed of well differentiated endometrioid carcinoma (EC) and undifferentiated carcinoma [1]. Contrastingly, carcinosarcoma (CS) is composed of high-grade carcinomatous and sarcomatous components, with the latter containing homologous and/or heterologous elements [1]. Identifying heterologous elements via immunohistochemistry (IHC) is helpful for differentially diagnosing DC and CS cases. However, in some cases, both the dedifferentiated part of DC and the sarcomatous part of CS exhibit similar nonspecific vimentin and keratin expression [2]. Grade 2 (G2) and Grade 3 (G3) EC cases also exhibit variable vimentin and keratin expression [3], indicating that the value of IHC for vimentin and keratin is limited in endometrial cancer diagnosis. Therefore, developing a novel integrated strategy combining histological and genomic analyses is necessary for the differential diagnosis of DC, EC, serous carcinoma (SC), and CS with areas of solid proliferation [4-6].

Next-generation sequencing (NGS) has recently become a standard procedure for cancer genomic analysis [7, 8]. This is largely due to the development of improved techniques, which allow the use of formalin-fixed paraffin-embedded (FFPE) tissues and liquid-based cytology specimens [9, 10]. We previously established a cancer gene panel comprising 60 genes and 17 microsatellite foci. This customized panel was used to analyze genetic profiles using FFPE tissues of endometrial cancers in terms of gene mutations, tumor mutation burden (TMB), and microsatellite instability (MSI) [11]. In this study, the above panel was further modified to detect *POLE* for evaluating gene alterations that can categorize DC, CS, SC, and EC with solid proliferation into MMR-deficient (MMR-d), p53 mutation (p53mut)-type, *POLE* mutation (*POLE*mut)-type, and cases with no specific molecular profile (NSMP) according to The Cancer Genome Atlas (TCGA) classification. Furthermore, we used an IHC panel containing mismatch repair (MMR) proteins (MLH1, PMS2, MSH2, and MSH6), p53, ARID1A, PTEN, vimentin, WT-1, estrogen receptor (ER), and cyclin D1 (CycD1), which is routinely available for FFPE tissue sections in pathology laboratories. This panel was used to determine whether an integrative approach utilizing IHC and NGS could aid pathologists in the differential diagnosis and histology-dependent classification of endometrial cancers.

## Materials and Methods

### Ethics Approval and Samples

All patients were registered at the Clinical Research of Cancer Gene Panel Analysis of Gynecologic Cancers Study, which was conducted from January 2019 to October 2021 at the Kagoshima University Hospital. The clinical samples used in this study were approved by the Ethics Committees for Clinical and Epidemiologic Research at Kagoshima University (approval number: 180215) and written informed consent was obtained from all participants. Among the 155 cases entered, 36 cases, including 16 G2 and 3 G3 EC cases, 3 DC cases, 4 CS cases, 9 SC cases, and 1 large cell neuroendocrine carcinoma (LCNEC) case with areas of solid proliferation were included in this study. Clear cell carcinoma (CCC) is considerably less frequent in the Gynecologic Cancers Study in our hospital, therefore, CCC cases were excluded from this study. No patients with undifferentiated carcinoma were included.

### Tissue Preparation and Diagnostic Criteria

Resected tissues obtained by hysterectomy were fixed with 10% neutral phosphate-buffered formalin, routinely processed for paraffin embedding, and sectioned for hematoxylin and eosin (HE) staining, IHC, and NGS. Pathological diagnoses were made according to the World Health Organization (WHO) classification system [1]. The following criteria were used for diagnosing SC: carcinoma showing complex papillary, glandular and/or solid growth patterns with marked nuclear pleomorphism; CS: admixture of high-grade müllerian type carcinomatous elements and apparent mesenchymal morphology, as determined using homologous (CD10, desmin, alpha-smooth muscle actin) or heterologous (myogenin, MyoD1, S-100) IHC markers [12, 13]; DC: biphasic tumor consisting of EC and undifferentiated nested or trabecular architecture with no gland formation; EC: glandular proliferation with smooth luminal outline, composed of columnar cells with pseudostratified nuclei. Grading criteria of EC: Grade 1, 2, and 3, respectively, exhibit ≤5%, 6–50%, and >50% solid growth was used.

The dedifferentiated parts of DC are positive for epithelial marker expression in scattered cells, and mesenchymal elements of CS are weakly positive or negative for these markers; hence, diffuse expression of keratins (AE1/AE3 and CAM5.2) and EMA was used to differentiate endometrial cancers [1].

### Criteria for Evaluating the IHC Results of MMR Proteins, p53, ARID1A, and PTEN

The antibodies used for IHC are listed in Online Resource 1. MMR-proficient (MMR-p) was defined as positive nuclear staining for all MMR proteins (MLH1, PMS2, MSH2, and MSH6). MMR-d was defined as the complete absence of nuclear staining for any MMR proteins [14]. p53 expression resulting in scattered nuclear staining with variable intensity was categorized as wildtype (wt p53) pattern, whereas diffuse and strong nuclear overexpression or a complete loss of expression was defined as mutation (mt p53) pattern. For ER, vimentin, WT-1, CycD1, ARID1A, and PTEN expression, the percentage of marker-positive areas assessed in the tumor section were evaluated in 10% increments. In areas of glandular or carcinomatous and solid proliferation, the expression of ARID1A and PTEN was categorized as very low at <10%, and as lost at <5%.

### Cancer Panel Design, DNA Isolation, and NGS Analysis

A cancer panel was redesigned by making a minor modification of the previous panel [11] to include 56 cancer-related genes and 17 microsatellite foci selected from the QIAseq Targeted DNA Custom Panel (Qiagen, Reston, VA, United States), with 2,640 primers for the regions of interest (194,131 bp) and an average exon coverage of 99.87% (Online Resource 2). Whole blood DNA was extracted using QIAamp DNA Blood Mini Kits (Qiagen). Cancer DNA was obtained from three to six sections (10 μm thickness) of FFPE tissues, representing more than 30% of the cancerous tissue. When cancer is composed of various histology, several tissue sections were subjected to NGS analysis if separate sampling of each element was possible by micro or macrodissection. The FFPE sections were first incubated with proteinase K (Promega, Madison, WI, United States) for 15 h at 70°C, followed by incubation at 98°C for 1 h in the lysis buffer (Promega). Following centrifugation (12,000 × g, 5 min at 4°C), the supernatants were applied to a Maxwell^®^ RSC DNA FFPE kit and the Maxwell 16 system (Promega). The concentrations of extracted DNA were measured using Qubit 3.0 dsDNA BR assay kits (Life Technologies, Grand Island, NY, United States), and the DNA quality was monitored using the QIAseq DNA quantiMIZE kit (Qiagen). DNA with a quality check score <0.04 was considered high-quality DNA [11]. NGS was performed using a MiSeq sequencer (Illumina, San Diego, CA, United States) as previously described [11].

Sequence data were annotated as previously described using the Qiagen Web Portal service (https://www.qiagen.com./us/shop/genes-and-pathways/data-analysis-center-overview-page/) and Mitsubishi Space Software (Amagasaki, Hyogo, Japan) [11, 15]. The COSMIC database and human genome reference GRCh37 (hg19) (https://www.ncbi.nlm.nih.gov/assembly/GCF_000001405.13/) were used as references. The sequence data obtained from whole blood DNA were used only as a reference, and germline analysis was not performed.

### Calculation of Copy Number Alteration

To calculate the copy number (CN) from the baseline data used for counting correction per amplicon, the number of reads sequenced in each amplicon was counted, and the reads per million (RPM) value was determined. The RPM coefficient of variation (CV), mean, and median value per amplicon in at least 100 FFPE samples were calculated. The RPM median of the amplicons with a CV < 0.34 and mean > 10 was set as the baseline. The number of reads sequenced in each 56‐panel amplicon was counted in the sample to calculate the CN of each sample. The baseline ratios {log2 ratio [ = log2 (sample RPM/baseline RPM median)]} in amplicons that satisfied the conditions of CV < 0.34 and mean >10 were counted, and the overall SD and median value of the log2 ratio for each gene was calculated. The genes with a log2 ratio median value >2 SD were categorized as amplified, while the genes with a log2 ratio median value <−2 SD were categorized as gene loss [16].

### Calculation of Cut-Off Values for TMB and MSI Scores

Missense mutations with more than 10% variant allele frequency, including nonsynonymous mutations and internal deletions, were counted as somatic mutations. The TMB was calculated as the number of single nucleotide variants million per base pairs (Mbp) of the DNA sequence [17, 18], and the MSI scores were determined using MSIsensor (ver. 1.0) [19, 20]. To determine the cutoff values of MSI and TMB using receiver operator characteristic curves, 59 samples from 41 endometrial cancer cases were used.

### Statistical Analysis

All values were expressed as the mean ± standard error. Significant differences were identified using student’s or Welch’s *t*-tests and were considered significant at *p* < 0.05.

## Results

### IHC Profiles

**FIGURE 1:**
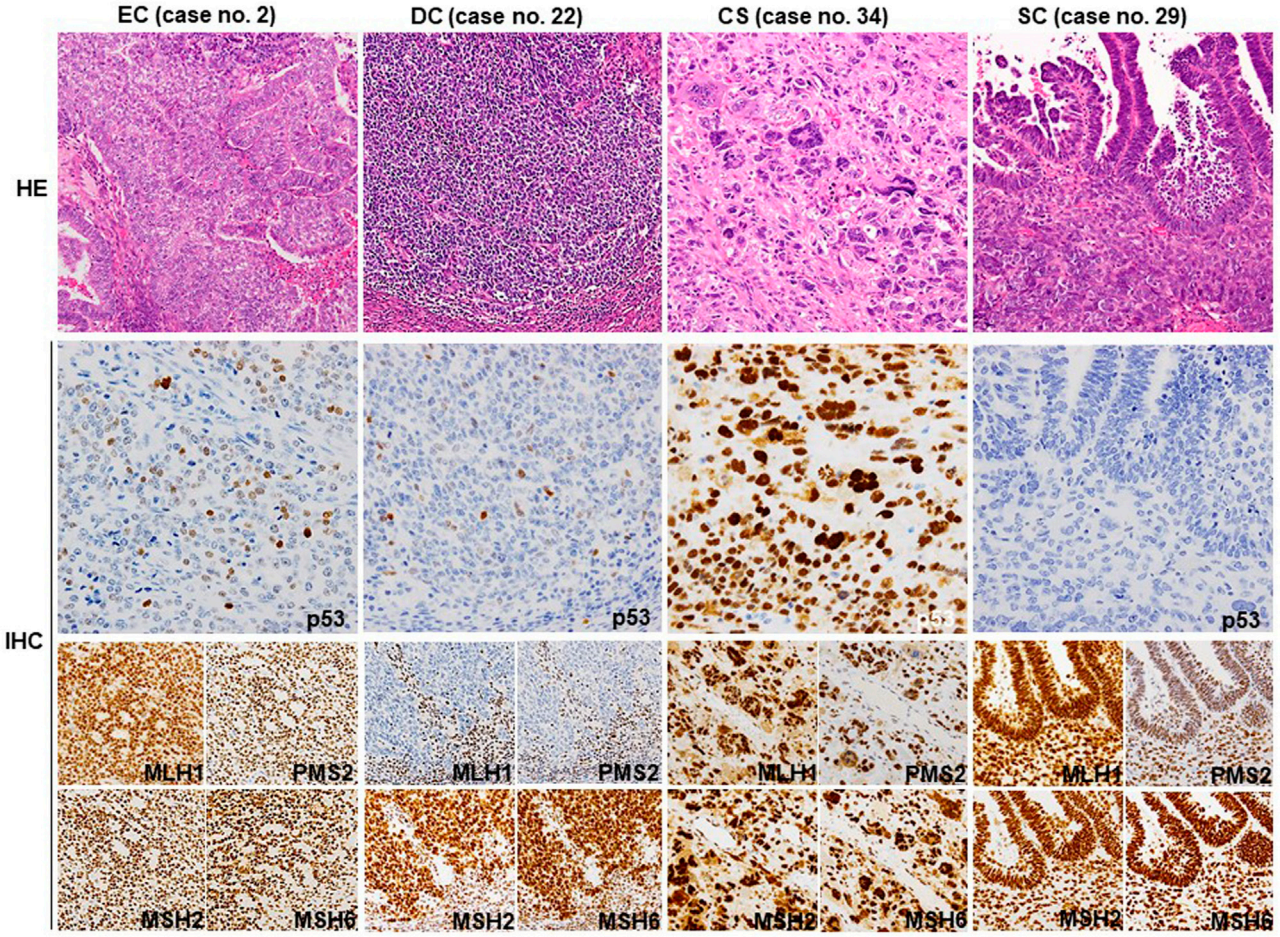
Representative histology and IHC of p53 and MMR proteins. Representative HE sections from G2 EC (no. 2) showing well differentiated glandular and less differentiated solid areas. A few p53-positive tumor cells are observed, indicating wt p53 expression. All four MMR proteins are diffusely positive. The dedifferentiated part of DC (no. 22) exhibits wt p53 expression pattern and loss of MLH1 and PMS2 expression. Stromal lymphocytes also show a positive reaction as an internal control. The sarcomatous element of CS (no. 34) shows diffuse staining for p53 and all four MMR proteins. SC (no. 29) shows complete loss of p53 expression in both glandular and solid elements. The MMR protein expression is well preserved. CS, carcinosarcoma; SC, serous carcinoma; HE, hematoxylin and eosin (Original magnification: ×200); IHC, immunohistochemistry. (Original magnification: p53 × 400, MMR ×200).

The IHC results regarding the MMR proteins, p53, ARID1A, PTEN, WT-1, ER, CycD1, and vimentin are summarized in Table 1 (extracted) and Online Resource 3 (detailed), and representative photomicrographs are shown in Figure 1. Among 19 EC cases, 16 showed the wt p53 pattern, and three showed the mt p53 pattern. Six EC and three DC cases showed loss of MMR protein expression. PTEN expression was very low (<10% area showing expression) or lost (<5% area showing expression) in all DC (3/3) and most EC cases (12/19), while ARID1A was lost less frequently in DC (1/3) and EC cases (7/19). All SC (9/9) and some CS (2/4) cases exhibited the mt p53 pattern in carcinomatous and sarcomatous elements. Three CS cases were MMR-p, whereas one was MMR-d. Almost all SC cases were MMR-p (8/9). PTEN expression was lost in one CS and five SC cases. ARID1A expression was well preserved in CS (3/4) and SC (8/9) cases. One LCNEC exhibited MMR-p and had mt p53 as well as diffuse PTEN and ARID1A expression. The expression of WT-1, vimentin, CycD1, and ER was variable in all carcinoma types. The genomic correlation between CycD1, ER, PTEN, and ARID1A is described later.

**TABLE 1:** Summary for histological diagnosis, expression of MMR proteins and p53, and genomic profile in 36 cases.

Case no.	Age	Histological diagnosis		MMR	p53	MSI	TMB	SNV and delins mutations								TCGA type
								*TP53*	*MLH1*	*PMS2*	*MSH2*	*MSH6*	*POLE*	*POLD1*	*TERT*	
1	60	EC (G2)		pro.	wt	Low	High									NSMP
2	54	EC (G1)		pro.	wt	Low	U-high			p.Val63Met		c.2389G>T	p.Val411Leu			POLEmut
		EC (G2)				Low	U-high					c.2389G>T	p.Val411Leu			
3	67	EC (G2)		pro.	wt	Low	Low									NSMP
4	68	EC (G2)		def.	wt	High	High									MMR-d
5	63	EC (G2)		pro.	wt	Low	Low									NSMP
6	64	EC (G3)		def.	wt	High	High	p.Arg202Cys								MMR-d
7	59	EC (G2)		def.	wt	High	High					p.Pro991Leu				MMR-d
8	55	EC (G2)		pro.	wt	Low	Low									NSMP
9	60	EC (G2)		def.	wt	High	High				p.Phe22fs					MMR-d
10	61	EC (G2)		pro.	wt	Low	Low									NSMP
11	78	EC (G3)		def.	wt	High	U-high	p.Arg175His				p.Met491fs				MMR-d
12	60	EC (G2)		pro.	wt	Low	Low									NSMP
13	55	EC (G2)		pro.	mt	Low	U-high	p.Arg213*		p.Arg107Trp p.Arg134Gln p.Arg427Cys	p.Asp758Asn	p.Arg298Gln p.Lys632Arg p.Glu641* p.Glu995Lys	p.Pro286Ser p.Val411Leu		p.Ser579Asn c.*334C>T	POLEmut
14	65	EC (G2)	gld.	pro.	mt	Low	Low	p.Arg273Cys								p53mut
			solid	pro.	mt	Low	Low									
15	68	EC (G2)	gld.	pro.	wt	Low	Low			c.903+2T>A						NSMP
			solid	pro.	wt	Low	High			c.903+2T>A						
16	55	EC (G3)		pro.	mt	Low	Low	c.993+1G>T								p53mut
17	73	EC (G2)	gld.	pro.	wt	Low	Low									NSMP
			solid	pro.	wt	Low	Low									
18	53	EC (G2)	gld.	def.	wt	High	Low		p.Glu613_Phe614del						p.Arg678Trp	MMR-d
			solid	def.	wt	High	U-high		p.Glu613_Phe614del					c.3582+5C>T	p.Arg678Trp	
19	56	EC (G2)	gld.	pro.	wt	Low	U-high				p.Glu483*	p.Arg1201Gln p.Glu1234*	p.Pro286Arg			POLEmut
			solid	pro.	wt	Low	U-high				p.Glu483*	p.Arg240Gln p.Glu1322*	p.Pro286Arg			
20	51	DC	EC (G1)			High	High							p.Asp289dup		MMR-d
			De	def.	wt	High	High		p.Ser388Phe					p.Asp289dup	p.Ala288Val	
21	57	DC	EC (G3)	def.	wt	High	High	p.Lys373fs							p.Ala288Val p.Trp371Cys p.Arg481Gly	MMR-d
			EC (G 1)			High	High		p.Asn287fs					p.Tyr581His	p.Trp371Cys	
			De			High	High	p.Lys373fs				p.Ala69Thr		p.Tyr581His	p.Trp371Cys	
22	71	DC	EC (G1)			High	High		p.Ser577Ser p.Trp712*							MMR-d
			De	def.	wt	High	High		p.Ser577Ser p.Trp712*					p.Asp1013fs		
23	64	SC		pro.	mt	Low	Low	p.Arg249Ser								p53mut
24	67	SC		pro.	mt	Low	Low	p.Gly245Ser p.Leu43fs								p53mut
25	57	SC		pro.	mt	Low	Low	p.Arg248Gln								p53mut
26	63	SC	Ad	pro.	mt	Low	Low	p.Ile255Ser								p53mut
			Spn			Low	Low	p.Ile255Ser								
27	72	SC		def.	mt	High	U-high	p.Arg306*						p.Arg352His c.2466+4C>T		p53mut/ MMR-d
28	75	SC		pro.	mt	Low	Low	p.Ser241Phe								p53mut
29	70	SC		pro.	mt	Low	Low	p.Gln144*								p53mut
30	65	SC		pro.	mt	Low	Low	c.817C>T								p53mut
31	77	SC		pro.	mt	Low	Low	p.Ala276Gly								p53mut
32	80	CS		pro.	mt	Low	Low	p.Asn239Ser								p53mut
33	58	CS		pro.	mt	Low	Low	p.Cys176Phe c.706T>A								p53mut
34	63	CS	Car	pro.	wt	Low	Low									NSMP
			Src			Low	Low									
35	64	CS	Car	def.	wt	High	High									MMR-d
			Src			High	High									
36	63	LCNEC		pro.	mt	Low	Low	p.Phe113Ser								p53mut

EC, endometrioid carcinoma; gld., glandular part; solid, solid part; DC, dedifferentiated endometroid carcinoma; CS, carcinosarcoma; SC, serous carcinoma; LCNEC, large cell neuroendocrine carcinoma; De, dedifferentiated part; Ad, adenocarcinoma part; Car, carcinomatous part; Sar, sarcomatous part; Spn, spindle cell part; MMR, mismatch repair protein; MSI, macrisatellite instability; TMB, tumor mutation burden; CNA, copy number alteration; Amp, amplification; SNV, single nucleotide variant; U-high, ultra high; pro., proficient; def., deficient; wt, wild-type; mt, mutation; PLOEmut, POLE, mutation; MMR-d, MMR-deficient; NSMP, no specific molecular profile; p53mut, p53 mutation.

### Cutoff Values of TMB and MSI Scores

**FIGURE 2:**
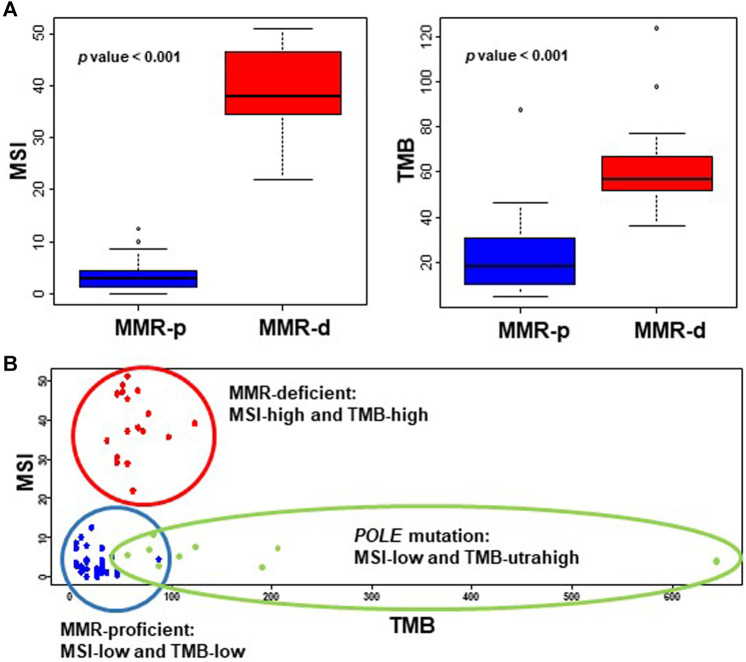
Values of MSI and TMB in endometrial cancers. **(A)** The MSI **(left panel)** and TMB **(right panel)** scores of MMR-d EC cases are significantly higher than those of EC cases that are MMR-p. **(B)** The MSI and TMB scores of endometrial cancers are plotted as a scattergram, showing clear distinction of MMR-p (blue dot) and MMR-d cases (red dot), and TMB-ultrahigh *POLE* mutation cases (green dot). MMR, mismatch repair; MSI, microsatellite instability; TMB, tumor mutation burden; MMR-p, mismatch repair-proficient; MMR-d, mismatch repair-deficient.

The TMB values of wildtype and mutated *POLE* cases were 36.3 ± 3.8 and 175.1 ± 61.0 (*p* = 0.026), respectively, and the cutoff value for TMB-ultrahigh (TMB-UH) was calculated as 72. Excluding the *POLE*mut cases, the TMB values of MMR-p and MMR-d cases were 22.5 ± 3.0 and 63.0 ± 5.1, respectively (*p* < 0.001), and the cutoff values for TMB-high (TMB-H) and -low (TMB-L) were calculated to be 42. Similarly, the MSI values of MMR-p and MMR-d cases were 3.6 ± 0.5 and 38.7 ± 2.0, respectively (*p* < 0.001), and the cutoff values for the differentiation of MSI-high (MSI-H) and -low (MSI-L) were estimated to be 13. The distribution of TMB and MSI scores of the tested endometrial cancers for cutoff value estimation is shown in Figure 2.

### Genomic Profile

Genomic profiles of 36 cases are shown in Table 1 (extracted) and Online Resource 4 (detailed). All three DC cases (no. 20–22) carried *MLH1* and *POLD1* mutations with TMB-H and MSI-H, and exhibited variable mutations in *PTEN*, *ARID1A*, *PIK3CA*, *PIK3R1*, and *CTNNB1*. The *TERT* coding region was mutated in two DC cases, although the promoter was not. All 19 EC cases exhibited common mutations in *PTEN*, *ARID1A*, *CTNNB1*, *PIK3CA*, and *PIK3R1*. Among the 19 cases, 16 cases (except for case no. 2, 13, and 19) included MMR-p and MMR-d cases and exhibited higher TMB scores than did the SC cases (Table 2). Six EC cases that were MMR-d had higher TMB and MSI scores than the ten MMR-p cases and were classified as TMB-H and MSI-H (Table 3). Three EC cases (no. 2, 13, and 19) that harbored a *POLE* mutation were classified as TMB-UH and MSI-L.

**TABLE 2:** MSI and TMB in each histological subtype.

	n	MSI score	EC	CS	SC
DC	3	39.2 ± 5.5	*p* = 0.037	Ns	*p* < 0.001
EC	16	16.5 ± 5.0		Ns	ns
CS	4	20.5 ± 10.2			ns
SC	9	7.9 ± 3.6			
	**n**	**TMB score**	**EC**	**CS**	**SC**
DC	3	68.7 ± 4.6	ns	*p* = 0.012	*p* = 0.013
EC	16	43.2 ± 6.9		Ns	*p* = 0.041
CS	4	23.2 ± 11.6			ns
SC	9	21.8 ± 9.9			

The MSI, and TMB, scores are presented as mean ± standrad error. EC with POLE, mutation is excluded; MSI, microsatelliete instability; TMB, tumor mutation burden; EC, endometrioid carcinoma; CS, carcinosarcoma; SC, serous carcinoma; ns, not significant.

**TABLE 3:** MSI and TMB in EC.

EC	n	MSI score	TMB score
MMR-p	10	2.3 ± 0.6	26.3 ± 3.5
MMP-d	6	40.4 ± 3.7	66.1 ± 12.8
*p* value		<0.0001	0.012

MSI, microsatellite instability; TMB, tumor mutation burden; EC, endometroid carcinoma; MMR-p, mismatch repair-proficient; MMR-d, mismatch repair-deficient.

Among the four CS cases (no. 32–35), two contained mutations in *TP53* and exhibited mt p53 expression. Furthermore, three CS cases, one with mutated and two with wildtype *TP53*, exhibited amplification of *MYC*, *CCNE1*, or *CCND1*. All CS cases were MMR-p (MSI-L and TMB-L) and showed no mutations in *MMR*, except for one case that exhibited a loss of MLH1 and PMS2 expression (MSI-H and TMB-H) without any mutations in *MLH1* and *PMS2*. IHC and NGS profiles of eight SC cases, displaying mt p53 expression and MMR-p characteristics were classified as MSI-L and TMB-L. One CS case (no. 32) showed *ERBB2* amplification. Among the eight SC cases, five showed amplification in *MYC*, *CCNE1*, or *CCND1*. One SC case (no. 27) exhibited MMR-d with wildtype *MMR* and was classified as TMB-UH and MSI-H. LCNEC (no. 36) exhibited an SC-like profile with mutations in *TP53* and *PIK3CA*. Other mutations such as *BRCA1*, *BRCA2*, and *ATM* were detected in some cases (nos. 2, 4, 5, 18, 19, 20, 22, 27, and 36) (Online Resource 4).

For the tumors composing of heterogenous elements, the genomic profile was evaluated separately for each element suing two or three FFPE sections in six cases of EC (no. 2, 14, 15, and 17–19), two of CS (nos. 34 and 35), one of SC (no. 26), and three of DC (nos. 20, 21, and 22). The genomic profiles were not exactly matched between the heterogenous elements, but were not so different as much as TCGA classification was revised (Online Resource 4).

### Correlation Between the Results of Genomic and IHC Analyses of p53, ARID1A, and PTEN

The correlation between the genomic and IHC analyses of ARID1A and PTEN is presented in Online Resource 5. Most CS (3/4) and SC cases (8/9) exhibiting high ARID1A expression (>90% of the positive area) had no mutations. Fourteen EC cases (14/19) harbored frameshift, nonsense mutations or splice variants. Among these, eight cases exhibited a loss of ARID1A expression (<5% of the positive area). PTEN expression was lost in one CS case with gene alterations (1/4). Although SC cases did not display *PTEN* mutations (9/9), five cases did not express PTEN. Among 19 EC cases, 18 carried *PTEN* mutations and 13 accompanied by a loss of PTEN expression (<5% of the positive area). As previously reported [21, 22], ARID1A expression was almost consistent with *ARID1A* mutations in EC, CS, and SC cases, and that of PTEN was consistent with *PTEN* mutations in EC cases. For CycD1 and ER, a correlation between genomic and IHC analysis results could not be established due to the limited number of cases exhibiting these gene mutations (data not shown). The status of *TP53* mutation matched well with the p53 IHC results in EC, SC, CS, and LCNEC cases (33/36), except for two EC cases (no. 6 and 11) and one DC case (no.21) that exhibited wt p53 IHC but harbored *TP53* mutations.

### TCGA Classification

Based on TCGA classification [23], EC cases were classified as *POLE*mut (3/19), MMR-d (6/19), NSMP (8/19), and p53mut (2/19), and all DC cases were categorized as MMR-d (3/3). CS cases were classified as MMR-d (1/4), p53mut (2/4), and NSMP (1/4). Most SC cases were classified as p53mut (8/9), and one was classified as MMR-d (1/9) (Table 4).

**TABLE 4:** Distribution of TCGA classification*.

	EC	DC	CS	SC	LCNEC
POLEmut	3	0	0	0	0
MMR-d	6	3	1	1	0
p53mut	2	0	2	8	1
NSMP	8	0	1	0	0
36	19	3	4	9	1

*Case no. 27, which exhibits both p53mut and MMR-d features, is categorized as p53mut type. EC, endometrioid carcinoma; DC, dedifferentiated edometrioid carcinoma; CS, carcinosarcoma; SC, seous carcinoma; LCNEC, neuroendocrine carcinoma; PLOEmut, POLE, mutation; MMR-d, mismatch repair-deficient; NSMP, no specific molecular profile; p53mut, p53 mutation.

## Discussion

Our study indicated that NGS-based genomic analysis using the custom-made small panel could be used to evaluate TMB and MSI and for the detection of gene mutations, thus aiding in the categorization of endometrial cancers with solid proliferation according to TCGA classification.

DC comprises well differentiated EC and undifferentiated carcinoma. Therefore, it is often difficult to differentiate DC from CS and EC. Differential diagnosis by HE staining alone is challenging when CS tissues exhibiting only subtle spindle cells or ambiguous heterologous sarcomatous elements, such as chondrosarcoma and rhabdomyosarcoma, and less evident serous morphology are involved [24]. Differentiation markers, such as myogenin, CD10, and desmin, are used to investigate the presence of heterologous or homologous sarcomatous elements in CS tissues. However, sarcomatous markers may be expressed focally even in carcinomatous areas [2, 3]. Moreover, the epithelial markers (keratins and EMA) were sometimes weakly expressed in the scattered cells of dedifferentiated elements of DC or absent in the sarcomatous parts of CS tissues [1]. Therefore, it may be difficult to differentiate between DC and CS, even with additional analysis using IHC. The effects of interobserver variability on the diagnosis of DC, CS, and EC are well established [25, 26]. Therefore, IHC results alone may be insufficient for distinguishing these cancers. Consequently, genomic analysis appears to be a more suitable option for developing an integrated strategy for the differential diagnosis of endometrial cancers [27-29].

We found that DC cases typically harbored mutations in *MLH1* and *POLD1* as well as exhibited variable mutations in *PTEN*, *ARID1A*, *PIK3CA*, *PIK3R1*, and *CTNNB1* with MSI-H and TMB-H. Contrastingly, most SC cases and some CS cases (MSI-L and TMB-L) carried *TP53* mutations, while *PTEN*, *ARID1A*, *PIK3CA*, *PIK3R1*, and *CTNNB1* mutations were less frequent. Since CS is a biphasic tumor with a carcinomatous element exhibiting high-grade müllerian type carcinoma [12, 13, 24], CS cases harbored a *TP53* mutation, similar to SC [30, 31]. MMR-d cases, which are mutually exclusive of *TP53* mutations [32, 33], are rarely observed in CS or SC (4–6%) [24, 30, 34-36]. As previously reported [37-39], our study also demonstrated that CS and SC cases typically exhibited amplification in *MYC*, *CCND1*, and *CCNE1*. These findings were distinct from those in DC cases.

Using IHC as a tool for p53 and MMR protein analysis may facilitate differential diagnosis of DC and CS, as demonstrated by a recent study [24]. Our results substantiated this finding and further elucidated the benefits associated with analysis of MMR proteins by IHC. However, some studies have indicated that CS exhibits a high rate of MMR-d cases (10–41%) [40, 41]. We propose that some DC cases, rather than true CS cases, may have been included in these studies, as many cases (>60%) demonstrated endometrioid morphology in the epithelial components [41]. Another study also demonstrated that DC cases were distributed in all TCGA classification categories [42]. The histological diagnosis of DC was based on the absence of E-cadherin expression and positive ZEB1 immunoreaction. These criteria were different from the diagnostic criteria used in our current study. In another report, a certain population of endometrial DC exhibited concurrent loss of ARID1A and ARID1B and loss of SMARCA4 or SMARCB1 expression [43]. Therefore, the use of the histological criteria and immunophenotyping in DC remains controversial.

EC comprises glandular and solid (>5%) areas diagnosed as G2 or G3 EC, based on the percentage of the solid components and nuclear atypia [44]. In EC, solid components are more evenly distributed and typically have indistinct borders between glandular parts. Meanwhile, dedifferentiated solid areas in DC are well demarcated from glandular parts [1]. However, the differentiation of EC with solid components from DC is sometimes difficult, especially when diagnoses are based on histological findings alone. MMR-d was shared between wildtype *POLE* EC and DC. As G2/G3 EC commonly exhibits *TP53* mutations secondary to *MMR* mutations [45], p53 IHC is not always useful for differentiating EC, DC, and CS from SC. Furthermore, like the SC case (no. 27) displayed both p53mut and MMR-d features, 3% of endometrial cancers exhibit other genomic features in addition to p53 abnormalities [45].

In accordance with previous studies [21, 22, 46], a high concordance was observed between ARID1A IHC and mutation status, while that between PTEN IHC and mutation was also high in EC but not in SC cases. Even IHC detects a greater population of PTEN loss than does NGS analysis [21], the frequency of SC cases showing loss of PTEN expression with no *PTEN* mutation might be unusually high in our study (five out of nine cases). The reported frequencies of SCs showing loss of PTEN expression range from 0 to 11% [47-50]. Since the method of PTEN IHC is often poorly reproducible [21, 51, 52], we cannot exclude a possibility of false negative PTEN IHC due to technical issues.

Compared with IHC-based analyses of MMR proteins, characteristic IHC profiles are not known for being used for the differential diagnosis of *POLE*mut EC from DC. The most characteristic feature of EC with *POLE*mut is ultrahigh TMB and low MSI scores [23]. However, to the best of our knowledge, a targeting antibody detecting mutated-POLE protein is unavailable, and no characteristic histological findings associated with *POLE*mut-type EC are presently known.

Overall, *MLH1*, *POLD1*, and *TERT* mutations in DC may be considered a characteristic molecular profile that can be used to distinguish it from EC. Pathogenic *POLD1* mutations have been observed in colorectal cancers and polymerase proofreading-associated polyposis syndrome [53]. Compared with *POLE*, *POLD1* mutations are less frequently observed in endometrial cancers, although a few *POLD1* mutations have been reported in G3 EC [54]. The sample size used to assess DC in this study was considerably small for determining the significance of *TERT* and *POLD1* mutations for tumor cell dedifferentiation in EC.

As endometrial LCNEC also demonstrates undifferentiated solid elements, LCNEC should be considered in the differential diagnosis of endometrial cancer with solid proliferation. However, LCNEC diagnosis is not challenging due to its distinct IHC profile [55]. Herein, LCNEC was MMR-p and exhibited mt p53 and diffuse ARID1A expression, and its NGS profile was similar to that of SC. Although a previous study indicated that LCNEC should be classified as a variant of DC [56], a recent report has indicated that the nature of endometrial LCNEC is heterogeneous, and molecular analysis has shown that LCNEC may be grouped under any TCGA classification [57].

In summary, the feasibility of using a small NGS cancer panel to facilitate the molecular categorization of endometrial DC, SC, CS, and EC with solid proliferation was investigated. Genomic analyses that detect alterations in TMB, MSI, and gene mutations including *MLH1*, *TERT*, *POLD1*, and *POLE*, IHC analyses that assess p53 and MMR protein expression, and other conventional IHC including PTEN and ARID1A exhibit significant potential as a practical diagnostic tool for determining endometrial cancer pathology.

## Data Availability

The raw data supporting the conclusions of this article will be made available by the authors, without undue reservation.
